# *Plasmodium vivax* and *Mansonella ozzardi* co-infection in north-western Argentina

**DOI:** 10.1186/1475-2875-12-248

**Published:** 2013-07-17

**Authors:** María J Dantur Juri, Cecilia A Veggiani Aybar, Eugenia S Ortega, Guillermina B Galante, Mario O Zaidenberg

**Affiliations:** 1 Instituto Superior de Entomología “Dr. Abraham Willink”, Facultad de Ciencias Naturales e Instituto Miguel Lillo, Universidad Nacional de Tucumán, Miguel Lillo 205, (4000) San Miguel de Tucumán, Tucumán, Argentina; 2 IAMRA, Universidad Nacional de Chilecito, 9 de Julio 22, (5360) Chilecito, La Rioja, Argentina; 3 Coordinación Nacional de Control de Vectores, Ministerio de Salud de la Nación, Güemes 125, Piso 1, (4400) Salta, Argentina

## Abstract

A case of co-infection with *Plasmodium vivax* and *Mansonella ozzardi* was detected in a blood sample from a person who had shown symptoms of malaria and lived in a city that was close to the Argentina/Bolivia border. The case was detected during a random revision of thick and thin smears from patients diagnosed with malaria from various towns and cities located in north-western Argentina between 1983 and 2001. Trophozoites of *P. vivax* were observed in the thin blood smear along with *M. ozzardi* microfilaria (larval form), which presented a long, slender, pointed anucleate tail and the absence of the sheath. This last characteristic is shared with *Mansonella perstans*, *Mansonella streptocerca* and *Onchocerca volvulus*. More rigorously controlled studies to detect other co-infection cases in the area as well as the possibility of importation from Bolivia into Argentina are currently ongoing. The relationship between the malaria parasite and microfilaria, the potential effect of malaria treatment on the development of *M. ozzardi*, and the possible impact of this microfilaria on the immunity of a person against *P. vivax* are all still unknown. This contribution constitutes a point of focus for future studies involving the interaction between the parasites and the potential risk that humans are exposed to.

## Background

Malaria caused by *Plasmodium vivax* spans the greatest geographic range [1]. Worldwide infections of *P. vivax* are estimated between 130 and 390 million, with 2.6 billion individuals living at risk of infection [1,2]. Severe and complicated malaria is generally caused by *Plasmodium falciparum*; however, an increasing number of *P. vivax* cases with severe manifestations have been reported recently [1,3]. It should also be noted that some researchers have cited cases of persons with malaria who do not present the typical symptoms caused by infection with *P. vivax* becoming asymptomatic patients [4-10].

The first cites of malarial disease in Argentina occurred between the end of the 19th Century and the beginning of the 20th Century and included reports on the geography of the disease within the country [11], the presence of both gametocytic and zygotic forms of *Plasmodium* parasites in *Anopheles* mosquitoes [12], and the parasitological, epidemiological and entomological conditions of malaria in north-western Argentina, recognizing the presence of “tropic malaria” (produced by *P. falciparum*), a “tertian malaria” (produced by *P. vivax*) and a “quartan malaria” (produced by *Plasmodium malariae*), with all types co-existing at the same time [13].

*Plasmodium vivax* was the only malaria parasite reported in the north-west region of the country since the 1970s [14-19]. Positive testing of blood samples for *P. vivax* was due to active searches for sick people conducted by technicians of the Ministry of Health of Argentina.

*Anopheles pseudopunctipennis* is the main malaria vector in north-western Argentina [14-16]. Malaria caused by *P. vivax* and transmitted by *A. pseudopunctipennis* is much more benign compared to infections caused by other malarial parasites; commonly observed manifestations include intermediate episodes of fever and chills.

Nematodes that cause filariasis have been reported throughout the tropical regions of the world [20-22]. Generally, they are found accidentally when patients with symptoms of malaria visit the physician and thick and thin blood smears reveal the presence of *Plasmodium* parasites with the larval forms of the nematodes called microfilaria. The presence of microfilaria infection in Argentina was recognized by malaria surveys in the north-western region of the country [23], with the species *Microfilaria tucumana* first described [24] followed by *Microfilaria dermaquayi*[13,25], a homonym of *Mansonella ozzardi*[26], being described later. The high prevalence in the Tucumán, Salta, and Jujuy provinces in north-western Argentina were reported by Mühlens *et al.*[13].

*Mansonella ozzardi* is endemic to the subtropical mountainous rainforest in the north-west region of Argentina [27]. In this region, transmission is related to ceratopogonid midges, *Culicoides lahillei* (main vector) and *Culicoides paraensis* (secondary vector) and black flies, *Simulium exiguum* (secondary vector) [28]. Although *M. ozzardi* is considered a relatively non-pathogenic filarial parasite, its pathogenicity is still a controversial subject requiring further study [29,30]. There is currently a lack of information about this disease, with the latest reports being those of Krolewiecki *et al.*[31] and Veggiani *et al.* (personal communication) on the influence of ivermectin in patients and the epidemiology of the disease in Argentina, respectively. A high prevalence of filariasis (20.7%) was observed in one locality in the north-west region of the country [27]. A similar result has been observed (a prevalence of 26.0%) in a rural community in the Bolivian Chaco region [32].

*Plasmodium* nematodes co-infection was widely reported in America [33-37]. Aráoz and Biglieri [23] and Rosenbusch [25] have cited numerous cases of people with microfilaria co-infection in the north-west region of Argentina. Later, Mühlens *et al.*[13] reported the finding of this microfilaria in blood smears with malaria parasites in the same region. Since this last paper, there were no reports of co-infections of *Plasmodium* microfilaria in the country; thus, the current study is the first report of the presence of *P. vivax* and *M. ozzardi* after several decades.

According to the World Health Organization (WHO) [19], Argentina is involved in the National Pre-Elimination Programme of Malaria with the aim to focus on the active detection of autochthonous cases. It has implemented several studies where the existence of co-infection of *P. vivax* with others parasites is considered important.

The present study aimed to detect co-infection with *P. vivax* and *M. ozzardi* in patients with a diagnosis of malaria who received anti-malaria treatment with primaquine-cloroquine in north-western Argentina from 1983 to 2001.

## Methods

### Case description

**Figure 1:**
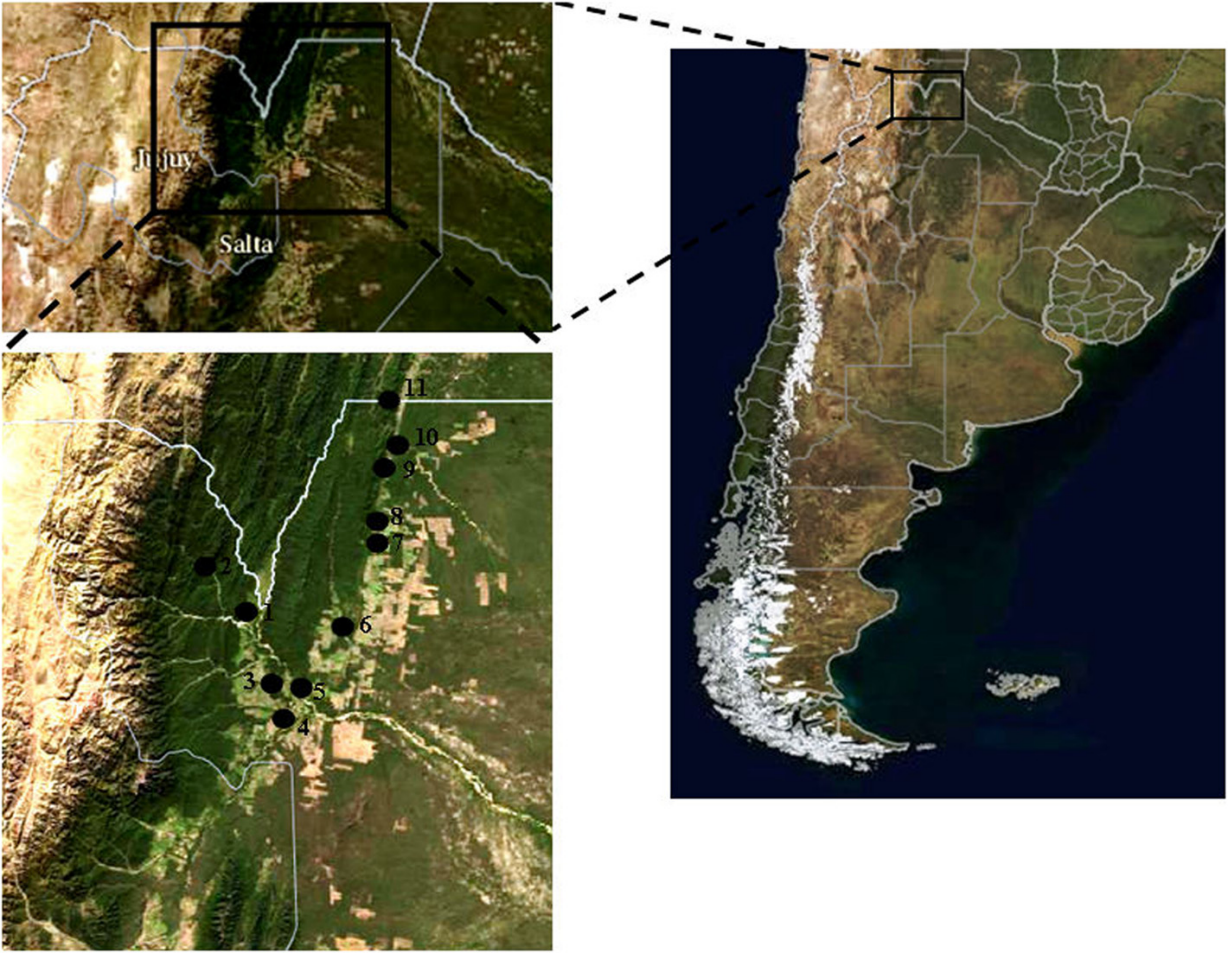
Geographical distribution of the localities in north-western Argentina (Aguas Blancas = 1, El Oculto = 2, San Ramón de la Nueva Orán = 3, Pichanal = 4, Embarcación = 5, General Ballivian = 7, General Mosconi = 8, Tartagal = 9, Aguaray = 10, Campo Durán = 11 and Salvador Mazza = 12).

From October to December 2012, it was performed a preliminary analysis of 166 thick and thin smears from patient blood samples from different towns and cities situated in north-western Argentina, at the border with Bolivia, where there are still reports of imported malaria cases. These towns and cities included Aguas Blancas (22° 43′ S; 64° 22′ W), El Oculto (23° 06′ S; 64° 30′ W), San Ramón de la Nueva Orán (23° 08′ S; 64° 20′ W), Pichanal (23° 19′ S; 64° 13′ W), Embarcación (23° 13′ S; 64° 06′ W) General Ballivian (22° 56′ S; 63° 52′ W), General Mosconi (22° 36′ S; 63° 49′ W) Tartagal (22° 32′ S; 63° 49′ W), Aguaray (22° 16′ S; 63° 44′ W), Campo Durán (22° 14′ S; 63° 42′ W) and Salvador Mazza (22° 04′ S; 63° 43′ W) (Figure 1).

**Figure 2:**
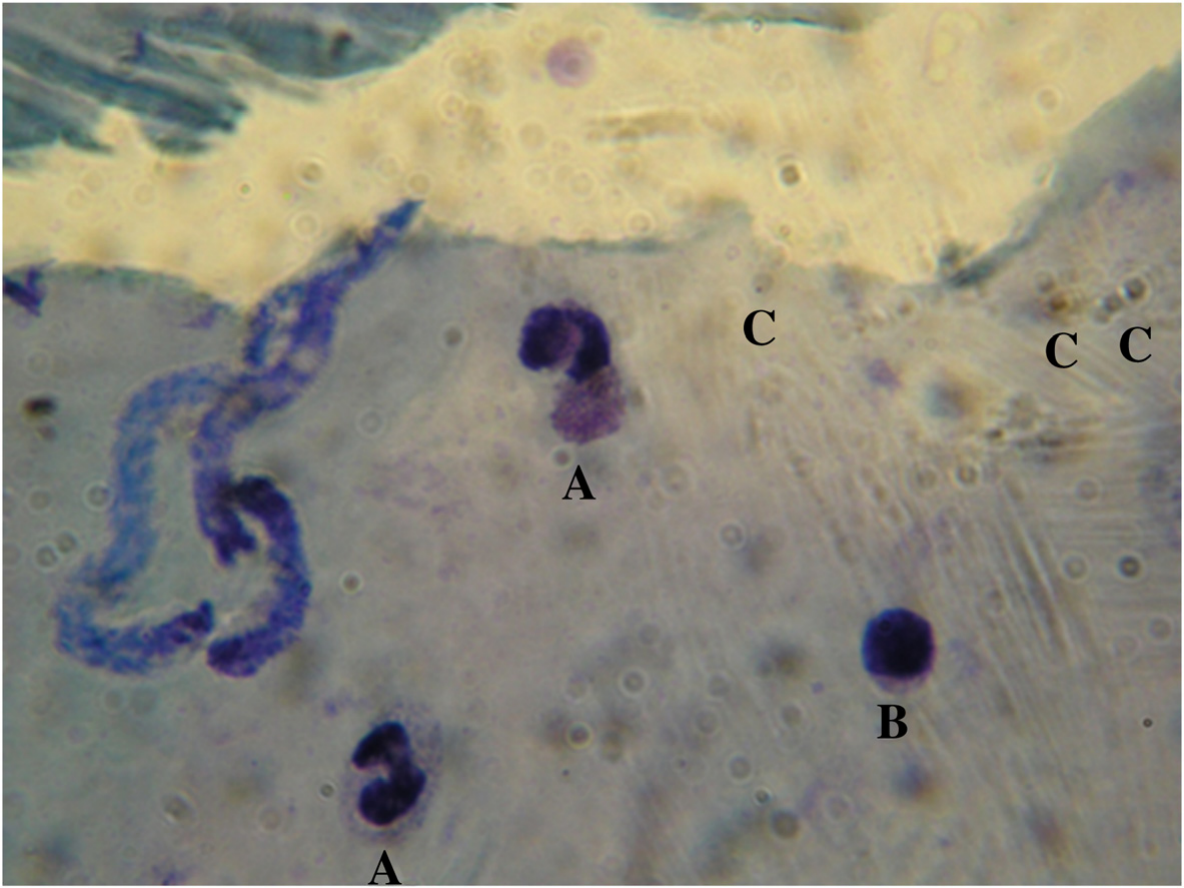
**Microscopic analysis of Giemsa-stained thin blood-smears from a patient infected with *Plasmodium vivax *and *Mansonella ozzardi *microfilaria parasites (A = neutrophils, B = lymphocyte and C = trophozoites).**

The reported case corresponds to a 70-year-old male living in the Tartagal locality, which belongs to the General José de San Martín Department, Salta province, north-west Argentina. The patient came to the regional malaria base with fever and chills. Microscopic revision was conducted on thick and thin smears, and parasites were visualized by using a binocular microscope with immersion lenses at 100% magnification. These tests revealed the presence of *P. vivax* - *M. ozzardi* parasites (Figure 2). The diagnosis was confirmed by a qualified specialist laboratory technician from the Reference Malaria Base of Salta City, Ministry of Health of Argentina.

The presence of *P. vivax* trophozoites was observed in the blood sample, showing an amoeboid cytoplasm and large chromatin dots. *Mansonella ozzardi* microfilaria appeared as described by Adami *et al.*[30] at the anterior region of the cephalic space that ends where the nuclear column begins, with an initial detailed nucleus followed by two that appear attached. The total length of the microfilaria was 148.2 μm and their diameter was 3.1 μm; these values were included in the averages established for this species.

An epidemiological sheet was completed with the microscopic analysis of blood samples, including information related to the consultation date (day, month, year), the patient’s name and address, place where the disease was supposedly acquired, travel during the previous three months, type of work, nationality and case classification. The epidemiological investigation revealed that the man living in Tartagal City and working in a banana farm close to the city acquired the malarial disease in Yacuiba (city on the Bolivia border) where he travelled two weeks before the report. The patient did not suffer any type of pain, with the presence of disease revealed by intermediate fever episodes. After laboratory confirmation, the treatment of the patient proceeded with a combination of chloroquine/primaquine pills for two consecutive weeks. The doses used were consistent with WHO guidelines [17] as follows: one tablet per day of chloroquine phosphate BP 242 mg (Micro Labs Limited, Hosur, India) for two weeks and one tablet per day of Aralen phosphate/primaquine phosphate 15 mg (Sanofi Aventis U.S. LLC, Bridgewater, NJ, United States) during three consecutive days at the start of treatment. At this time, treatment for the filariasis parasite was not considered, and no complications were reported during or after the malaria treatment.

### Consent

The study methodology, including the ethical aspects, was approved by the Ministry of Health of Argentina, which has an Ethical Committee who revised these aspects. They are included in the Protocol of the Manual de Normas y Procedimientos de Vigilancia y Control de Enfermedades de Notificación Obligatoria, Ministerio de Salud Argentina.

## Discussion

In north-western Argentina, the diagnosis of malaria is directly related to intermediate fever episodes, but the diagnosis of filariasis is a consequence of blood smear analysis for malaria. The first reports of malaria in Argentina showed that the disease was the most important parasitic disease of the time, not only because of the number of cases reported but also because of their wide geographical distribution across the country. The majority of malaria reports cited *P. vivax* as the most abundant parasite that appeared in blood smears, as well as the high prevalence of co-infection with *M. ozzardi*[26].

After the malaria eradication programme in 1959, malaria cases decreased considerably before a resurgence in disease in 1967 [38]. Since 1967, the active search for malaria patients by technicians of the Ministry of Health of Argentina, with adequate primaquine/chloroquine treatment, and the spraying in dwellings of the mosquito vector with 2.5% deltamethrin has reduced the incidence of malaria cases. The latest research indicates that the few autochthonous malaria cases were positive for *P. vivax.* This species seems to be the only parasite incriminated in malaria in human beings, and the most tolerant to the climatic and environmental changes, enabling its survival during this time in north-western Argentina [17-19,39]. The number of malaria cases with *M. ozzardi* co-infection also decreased dramatically during the last few decades, with the latest studies reporting high prevalence of microfilaria only within isolated communities, but affecting both sexes and with increasing infection rates progressively with increasing patient age [27,40-42].

## Conclusion

From the present report, it has concluded that further studies are necessary to search for cases of co-infection with *P. vivax* and *M. ozzardi*. These studies should try to establish the prevalence of co-infection and quantify the potential effects of malaria treatment on the development of *M. ozzardi* and the impact of this microfilaria on the immunity of humans infected with *P. vivax* in north-western Argentina. The results presented in this study could be used as the basis for future studies involving the interaction of these parasites and include other localities on the Bolivian border, considering that the parasites and their vectors do not recognize geographical barriers to their transmission.

## Competing interests

The authors declare that they have no competing interests.

## Authors’ contributions

MJDJ is a Research Assistant of CONICET and a Consultant of the Ministry of Health, this research is part of the studies that are ongoing in the country included in the Malaria Pre-elimination Phase according to the World Health Organization. She conceived the study and drafted the manuscript. CAVA checked all the human samples and also prepared the manuscript. ESO and GBG reviewed the literature and references. MOZ as a part of Ministry of Health of the Argentina contributed with the human samples to be analysed and participated in the edition of the manuscript. All authors read and approved the final manuscript.
